# Risk factors for pregnancy among rural and urban-dwelling adolescents in a high adolescent-pregnancy setting; a case control study

**DOI:** 10.4314/ahs.v26i2.14

**Published:** 2026-06

**Authors:** Nicolette Nabukeera-Barungi, Theresa Piloya-Were, Fatuma Namusoke, Joseph Rujumba, Flavia Namiiro, Lorraine Oriokot, Paul Mubiri, Sabrina Bakeera-Kitaka

**Affiliations:** 1 Makerere University College of Health Sciences, Department of Paediatrics and Child Health; 2 Makerere University College of Health Sciences, Department of Obstetrics and Gynaecology; 3 Mulago National Referral Hospital, Kampala Uganda; 4 Makerere University College of Health Sciences, School of Public Health

**Keywords:** Adolescent, Pregnancy, determinants, rural, urban

## Abstract

**Background:**

In Uganda, 1 in 4 girls has been pregnant by 18 years. This was worse during the COVID 19 pandemic. We determined risk factors for pregnancy among rural and urban dwelling adolescents in Uganda.

**Methods:**

We conducted a mixed-methods study among girls aged 13 to 19 years in a rural and urban district in Uganda. Quantitatively, we used an unmatched case control design involving 200 adolescents with pregnancy experience and 400 controls with no pregnancy experience. Quantitative data was analyzed using STATA version 14. Qualitative methods included 17 focus group discussions, 10 in-depth interviews and 20 key informant interviews with key stakeholders. Transcribed audio recordings were analyzed using a content thematic approach using Nvivo.

**Results:**

Of the 600 girls, mean age was 16.9 years (SD 3.84). Independent risk factors for pregnancy were age, out-of-school, a friend with teenage pregnancy, cigarette smoking, working for money and correct contraceptive knowledge. Protective factors included positive personal attitudes, father's education and larger household size. Qualitatively, inadequate information on sexual and reproductive health, early sexual debut, alcohol and drug abuse, absence of parental supervision, negative peer influence and poverty were reported.

**Conclusion:**

Overall, individual characteristics, behaviors and inadequate information increased the risk of pregnancy.

## Introduction

Adolescents are particularly vulnerable to sexual exploitation and high-risk behaviors which may result in pregnancy. A World Health Organization (WHO) report in 2014 put the global adolescent birth rate at 49 per 1000 girls aged 15 to 19 years^1^. Adolescent pregnancy in Africa and sub-Saharan Africa was 18.8% and 19.3% respectively^2^. In Uganda, the prevalence of teenage pregnancy is reported to remain high at 25% as the national prevalence rate^3^,^4^. The highest rates of adolescent pregnancy in Uganda are reported in the East Central region at 30.6% and rural teenagers having higher rates than their urban counterparts (25% verses 21%)^4^. Adolescent pregnancy is a major public health and social problem which has implications on the educational opportunity, population growth and health of women. Risk factors for teenage pregnancy include family and individual characteristics of the adolescents^5^.

Adolescent pregnancy results in various health complications for them and their infants, risk of stopping education^6^, early marriage, a lifetime cycle of poverty and deaths from pregnancy-related conditions^3^. Dropping out of school puts them into the cycle of poverty and this affects attainment of SDG 1 which calls for an end to poverty in all its manifestations by 2030.

Despite several efforts, adolescent pregnancy increased from 24% to 25% between 2011 and 2016^3^,^4^. Moreover, the current average rate of reduction in teenage pregnancies is so slow at 3% per year over the last 10 years. The 14% target of adolescent pregnancy by the Ugandan Ministry of Health^7^ may not be achieved unless there are efforts to accelerate the current reduction rate. We therefore set out to determine the risk factors for pregnancy among adolescents in Wakiso and Kamuli districts, an urban and rural district respectively.

## Methods

### Study design and setting

A concurrent mixed methods design utilizing both quantitative and qualitative data generation methods was used. Quantitatively, we used an unmatched case – control design to determine the risk factors of pregnancy among adolescents. Cases were currently pregnant or experienced a pregnancy. Controls were non-pregnant adolescents aged 13-19 years. We used in-depth interviews (IDI), focus group discussions (FGD) and key informant interviews (IDI) to explore risk factors for pregnancy.

Wakiso district is situated in the central region of Uganda near the capital city. The district has a population of 2,735,100 people, and 75% of it is urban. About 11% of females 10-19 years had ever been married and 14.7% had ever given birth in this district^8^. On the other hand, Kamuli district is rural, located in the eastern region of Uganda which has high adolescent pregnancy levels at 30%^4^. It has a population of 486,319 people. Here, 12.7% females aged 10-19 years had ever been married and15.8% had ever given birth^8^.

### Study Population

The study population included girls 13-19 years residing in Wakiso and Kamuli districts. For the qualitative design, we included adolescents, their parents/guardians, school teachers, health workers and other stakeholders in the same districts. The sample size was determined using a study on predisposing factors of teenage pregnancy in Uganda^9^. Using results from this study, we calculated the required sample size setting α=5% level of significance (95% certainty) and power of 80% (β = 0.2) for two controls per case, to be 100 cases and 200 controls. A total of 600 adolescents were interviewed (300 per district). The sample size for the qualitative part of the study was indicative, interviews continued until saturation of data.

### Sampling procedure

A two-stage cluster sampling approach was employed to select participants for quantitative component of the study. In the 1st stage of sampling districts were divided into clusters. In the 2nd stage, within each district, a probability proportional to size (PPS) sampling of 20 villages or enumeration areas (clusters) were taken. In each district, an up-to-date list of villages or enumeration areas with their sizes (number of households in each) was obtained. PPS was done in Stata to obtain 20 clusters/villages for each district. Within each sampled cluster (village), 15 adolescents were selected of whom 5 were pregnant and 10 non-pregnant (controls). The controls were sampled using systematic random sampling. From the center of the village, every 5th house was visited by research assistants who took opposite directions. If they did not have an eligible adolescent, the next house was taken until an adolescent was enrolled. Cases were identified by the Village health teams (VHTs) probing the pregnancy status of all the adolescent girls, asking the health facilities in the community, parents and community leaders. Five of the pregnant girls identified from each village were randomly selected as cases. Respondents of the qualitative design were purposively selected.

### Data collection

Data collection took three weeks starting in June 2020 amidst the COVID 19 pandemic. The country was under partial lock-down and all schools were closed. VHTs and local village leaders led the research assistants into the community. Once in a selected household, the interviewers obtained written informed consent for those aged 18 years and above. If the respondent was under 18 years, parental consent was sought and assent was obtained from the adolescent before enrolment of the adolescents.

The dependent variable was adolescent pregnancy. Independent variables included socio-demographic variables like age of the girl in years, tribe, religion, school status and level of education, birth order, place of residence (classified as urban or rural), parental characteristics etc. We also collected data on Sexual and Reproductive Health (SRH) knowledge, behavior and attitudes of adolescents. Data on SRH knowledge was collected using an instrument based on “Illustrative Questionnaire for Interview-Surveys with Young People,” a core set of instruments endorsed by the World Health Organization (Cleland J, 2001). It had a set of questions and scores were allocated according to responses. These variables were collected using a structured questionnaire which was administered by youthful interviewers who had a university degree and were trained before data collection.

Qualitative methods were conducted using appropriate guides with open- ended questions to generate discussion. We conducted FGDs for various categories of adolescents and parents/caregivers in each of the 2 districts conducted by the investigators or research assistants. We observed FGD norms whereby the discussions were guided by an FGD guide, a moderator and an observer who took additional notes. The discussions took place in the appropriate local languages. In-depth interviews were conducted with adolescents involved in sensitive SRH issues. They included pregnant or lactating adolescents, any who used health services for STI services, HIV testing or contraceptive services. The key informants comprised of various stakeholders including donor community, adolescent health implementing partners, district and community leaders, health workers, counselors, village health team members and school teachers. All the qualitative discussions were audio recorded.

### Data analysis and management

Six investigators supervised all data collection to ensure quality. Two experts in qualitative data methods focused on the qualitative while the PI and 3 others focused on the quantitative data collection supervision in the field. Data was entered using Epi Info and later transferred to Stata V.15 for analysis. Data was summarized into frequencies, proportions and totals for categorical data while means, standard deviations and medians with interquartile ranges for continuous variables. The Mantel-Hansel and conditional logistic regression model was used to determine risk factors at univariate and multivariable analyses. Factors with p value ≤0.2 were considered for multivariate analysis using linear or Poisson models. All models were tested for multicollinearity. A relative wealth index was constructed using principal component analysis from a set of seven questions relating to household assets The index was divided in quintiles^10^.

Qualitative analysis involved transcription of all audio recordings. After this, transcripts from FGDs and IDIs in local languages were translated to English. A codebook with code definitions was developed before coding. Transcripts were then exported into Nvivo 11 for electronic coding and analysis using content thematic approach 11 and guided by the Social Ecological Model (SEM) model 12,13 to locate risks of adolescent pregnancy. Quotes to support the codes were put under each code. The codes were combined into broader themes. The resultant themes were displayed and compared across study groups (adolescent girls, their caregivers and key informants) through text descriptions and matrices.

### Ethical approval and consent to participate

The study obtained Ethical approval from Makerere University School of Public Health Research and Ethics Committee (HDREC 768). In addition, approval was sought from the Uganda National Council of Science and Technology (SS 5236). Administrative permissions were received from the District Health Officers of Wakiso and Kamuli. Furthermore, written informed consent from participants aged 18-19 years and assent from the participants below 18 years were obtained while ensuring privacy and confidentiality.

## Results

### Sociodemographic characteristics of the adolescents

Our study involved 600 girls whose mean age was 16.9 years (SD ± 3.84). To note, 9.7% were married or separated at the time of the study and 75% were school-going. Since 63% were aged 17-19 years, we used weighted percentages to cater for the uneven age distribution as shown in table 1 and 2. Other baseline characteristics of the adolescents are summarized in table 1 below.

**Table 1:** Socio-demographic characteristics of the adolescent surveyed (n=600)

Characteristic		Frequency	Weighted Percentages
**Age (years)**	13-14	85	15.3
	15-16	125	21.3
	17-19	390	63.3
**Marital status**	Single – not in relationship	340	57.6
	Single - in a relationship	175	32.7
	Married/separated	85	9.7
**Ever attended school**	Yes	586	98.7
	No	14	1.3
Highest level of education completed (n=586)**	None	153	34.4
	Primary	310	53.0
	Secondary and above	123	12.5
**School going**	Yes	430	75.3
**Religion**	Catholics	170	19.4
	Anglican	196	39.3
	Pentecostal	90	15.7
	Muslim	137	24.3
	Other	7	1.3
**Orphanhood status**	Both parents alive	459	77.0
	Both parents dead	22	2.7
	Only mother alive	81	15.0
	Only father alive	38	5.3
**Disability status**	Yes	48	11.0
	No	552	89.0
**Relationship to household head**	Spouse/Partner	83	9.3
	Daughter	401	76.6
	Other relatives	116	14.0

** Highest level completed – None–did not complete primary school or never went to school at all, Primary – completed Primary school and has a certificate, secondary – attained a certificate to complete secondary school and above

**Table 2:** Behaviour and experiences of the 600 adolescents

Covariates	Frequency	Weighted percentage
Ever had sex	337	62.3
Used a condom (N=337)	139	47
Reports being physically abused	116	16.7
Reports Sexual abuse	39	4.3
Has a friend who has ever got pregnant	339	56.0
Ever tested for HIV	323	54.0
Ever used contraceptives	106	21.0
Has a friend who uses drugs/alcohol/smoke	105	12.3
Ever taken alcohol	60	5.3
Ever smoked cigarettes	8	1.3
Ever used drugs	7	1.3
Have Freedom to clubs/dances	95	15.7
Ever attempted to take own life	153	26.3
Feels safe at home	495	81.0
Ever missed school due to menstruation	201	46.0
Does some work for money	131	17.0

Only 282 (42%) reported ever having discussed sex with their parents. Of the 600 adolescents, 240 (43%) reported their parents' parenting styles as being disciplinarian/authoritative, 249 (37%) reported their parents as being permissive/liberal. We also described the behaviors and experiences of adolescents which affect adolescent pregnancy and table 2 below has a summary of the findings. From the 200 girls who had ever been pregnant, the mean number of pregnancies was 1.1 (SD 0.64) and mean age of first pregnancy was 16.6 years.

### Bivariate analysis of the risk factors of adolescent pregnancy

At bivariate analysis, older adolescents, those who were married, school status, father's education, orphanhood status and family size were found to be statistically significant as shown in table 3 below.

**Table 3:** Bivariate analysis of socio-demographic risk factors for adolescent pregnancy

Characteristic		Currently pregnant N=200		Never been pregnant N=400	Chi-square	p-value
**Age group (years)**	13-14	1.0	20.7		91.63	<0.001*
	15-16	8.0	27.3			
	17-19	91.0	52.0			
**Marital status**	Single – not in relationship	30.5	69.7		143.38	<0.001*
						
	Single - in a relationship	33.0	27.2			
						
	Married/separated	36.5	3.0			
**Highest education completed (n=586)**	None	31.1	23.6		3.86	0.145
	Primary	49.0	54.9			
	Secondary and above	19.9	21.5			
**In school (n=547)**	Yes	52.7	89.8		94.13	<0.001*
**Orphan hood status**	Both parents alive	69.0	80.2		10.07	0.018**
	Both parents' dead	4.0	3.5			
	Only mother alive	18.5	11.0			
	Only father alive	8.5	5.2			
**Fathers highest level of education**	None	20.0	17.5		17.59	0.001*
	Primary	20.0	14.7			
	O-Level	9.0	13.5			
	A-level+	6.5	17.0			
	Don't know	44.5	37.2			
**Household size**	1-3	32.0	10.2		47.63	<0.001*
	4-6	29.5	37.0			
	7-8	15.5	28.5			
	9+	23.0	24.2			

Table 4 summarized the family and behaviour risk factors for adolescent pregnancy at the bivariate analysis. Many of the risky behaviours like alcohol use, pornography, freedom, as well as experiences of peers were risk factors for adolescent pregnancy. Surprisingly, “Knowledge of pregnancy prevention” and contraceptive use were statistically significant risk factors of pregnancy.

**Table 4:** Bivariate analysis of Family and Behaviour Risk factors for adolescent pregnancy

Characteristic			Currently pregnant	Never been pregnant	Chi-square	p-value
			N=200	N=400		
Parents are separated/divorced		Yes	36.5	28.0	4.52	**0.032**
		No	63.5	72.0		
Parenting style	Disciplinaria/Authoritarian		35.0	42.5	5.71	0.127
	Permissive or liberal		42.5	41.0		
	Uninvolved		9.0	8.2		
	Others		13.5	8.2		
Parent/s drink alcohol		Yes	26.0	18.7	4.35	0.118
		No	72.0	78.5		
		Don't know	2.0	2.7		
Sexual abuse experience		Yes	9.0	5.2	3.08	0.079
		No	91.0	94.7		
History of mental illness		Yes	5.5	2.7	2.85	0.091
		No	94.5	97.3		
Ever discussed sex with a parent		Yes	51.5	44.7	2.44	0.118
		No	48.5	55.2		
A friend who has ever gotten pregnant		Yes	75.0	47.3	41.78	<0.001*
		No	25.0	52.7		
Ever used contraceptives		Yes	34.5	9.2	58.44	<0.001*
		No	65.5	90.7		
Ever taken alcohol		Yes	14.0	8.0	5.33	0.021**
		No	86.0	92.0		
Ever smoked cigarettes		Yes	3.0	0.5	6.33	0.019**
		No	97.0	99.5		
Used drugs before		Yes	2.5	0.5	4.62	0.044*
		No	97.5	99.5		
Has freedom to go out to clubs/dances		Yes	21.5	13.0	7.23	**0.007** *
		No	78.5	87.0		
Feels safe at home		Yes	79.5	84.0	1.87	0.171
		No	20.5	16.0		
Owns a mobile phone		Yes	52.0	29.0	30.37	<0.001*
		No	48.0	71.0		
Has ever used pornography		Yes	61.5	45.5	13.66	<0.001*
		No	38.5	54.5		
Works for money		Yes	31.5	17.0	16.42	<0.001*
		No	68.5	83.0		
Knowledge of pregnancy prevention*			5.2 (1.2)	4.8 (1.3)	-2.99	0.003*
Positive personal attitudes*			3.9 (1.6)	4.4 (1.5)	4.39	<0.001*
Knowledge on contraceptive use*			3.3 (1.3)	2.7 (1.3)	-5.42	<0.001*

* Mean Scores (standard deviation) on SRH knowledge

### Multivariate analysis of the Risk Factors of Adolescent Pregnancy

All the variables with p-values below 0.2 were put in the model to determine independent predictors of adolescent pregnancy. Factors that are independent predictors of adolescent pregnancy included are summarized in Table 5.

**Table 5:** Multivariable logistic model for risk factors of adolescent pregnancy

Risk factor		Crude OR (95% CI)	Adjusted OR (95% CI)
Age		1.91 (1.68-2.16) *	1.65 (1.44 – 1.91) ***
Currently in school	Yes	1	1
	No	7.89 (5.02 – 12.38) ***	4.42 (2.57 – 7.60) ***
Orphan hood status	Both parents alive	1	
	Both parents' dead	1.32 (0.54 – 3.24)	
	Only mother alive	1.95 (1.21 – 3.16) **	
	Only father alive	1.88 (0.96 – 3.68) ***	
Fathers highest level of education	None	2.98 (1.47 – 6.08) **	3.06 (1.36 – 6.87) **
	Primary	3.55 (1.73 - 7.26) **	4.14 (1.73 – 9.89) **
	O'Level	1.74 (0.78 – 3.87)	1.79 (0.69 – 4.61)
	A'level+	1	1
	Don't know	3.12 (1.63 – 5.98) **	2.96 (1.36 – 6.42) **
Mother's highest level of education	None	2.02 (0.95 – 4.29) ***	
	Primary	2.26 (1.06 – 4.83) **	
	O'Level	1.46 (0.65 – 3.24)	
	A'level+	1	
	Don't know	2.0 (0.96 – 4.17) ***	
Household size	1-3	1	1
	4-6	0.25 (0.15 – 0.42) *	0.48 (0.24 – 0.96) **
	7-8	0.17 (0.09 – 0.30) *	0.35 (0.17 – 0.70) **
	9+	0.30 (0.18 – 0.51) *	0.61 (0.30 – 1.25)
Parents are separated/divorced	No	1	
	Yes	1.47 (1.02 – 2.12) **	
Parent drink alcohol	No	1	
	Yes	1.51 (1.01 – 2.27) **	
	Don't know	0.79 (0.25 – 2.53)	
Sexual abuse	No	1	
	Yes	1.78 (0.93 – 3.43) ***	
History of mental illness (parents)	No	1	
	Yes	2.06 (0.87 – 4.83) ***	
A friend who has ever got pregnant	No	1	
	Yes	3.35 (2.30 – 4.87) *	
Ever used contraceptives	No	1	
	Yes	5.17 (3.30 – 8.08) *	
Ever taken alcohol	No	1	
	Yes	1.87 (1.09 – 3.21) **	
Ever smoked cigarettes	No	1	1
	Yes	6.15 (1.23 – 30.81) **	6.16 (1.51 – 25.13) **
Used drugs before	No	1	
	Yes	5.10 (0.97 – 26.57) ***	
Freedom to club/dance	No	1	
	Yes	1.83 (1.17 – 2.86) **	
Ownership of mobile phone	No	1	
	Yes	2.65 (1.86 – 3.77) *	
Ever used pornographic materials	No	1	
	Yes	1.91 (1.35 – 2.71) *	
Works for money	No	1	
	Yes	2.24 (1.51 – 3.33) **	
Correct Knowledge of pregnancy prevention		1.23 (1.07 – 1.42) **	
Positive personal attitudes		0.78 (0.69 – 0.87) *	0.85 (0.73 – 0.99) **
Correct Knowledge on contraceptive use		1.45 (1.26 – 1.66) *	1.29 (1.08 – 1.53) *
N=547, Pearson chi2 (491) = 426.83, p-value = 0.983			

*** p<0.001

** p<0.05

* p<0.10

We conducted a sub-analysis by urban/rural and found some differences in adolescent pregnancy as shown in table 6 below. In both districts, age and “not going to school” were risk factors for pregnancy. In Kamuli district, sexual abuse, working for money, “ever-taken alcohol” and accurate knowledge of contraceptives increased the risk. In Wakiso district, “ever wanted to take her life”, no education and poverty were risk factors.

**Table 6:** Multivariate analysis of Predictors of adolescent pregnancy by district

Characteristic	Characteristic	Kamuli (RURAL)	Wakiso (URBAN)
		Adjusted OR (95%CI)	Adjusted OR (95%CI)
Age		1.71(1.40–2.07)***	2.12(1.66–2.71]***
Not going to school		6.13(2.91–12.92)***	2.27(1.01–5.10)**
Sexual abuse		4.09(1.52–10.99)**	
Ever taken alcohol		3.19 (1.16–8.81)**	
Feels safe at home		0.42 (0.18–0.99]**	
Works to get some money		2.64(1.20–5.78]**	
Positive personal attitudes		0.76(0.61–0.94)**	
Accurate Knowledge on contraceptives		1.31 (1.00–1.71]**	
Highest level of education completed	None		1
	Primary		0.311(0.12–0.80)**
	Primary+		0.13 (0.04–0.42)**
Social economic status	Poor		1
	Moderate		0.77(0.35–1.68)
	Rich		0.28(009–0.84)**
Ever wanted to tale own life	Yes		1
	No		3.07(1.42–6.64)***

### Qualitative Findings

Qualitative findings revealed factors that protect or increase risk of adolescent pregnancy and these are summarized in matrix 1.

**Matrix 1:** Protective and risk factors for teenage pregnancy

Level	Protective factors against pregnancy	Factors increasing pregnancy
Individual	- Being in school - Having personal goals or aspirations - Access to SRH information - Use of family planning - Religion/fear of God	- Inadequate information on SRH - Early sexual debut - Alcohol and drug abuse - Being out of school - Older adolescents
Relationship	- Good relationship with parents - Parent-adolescent communication - Parental guidance and supervision - Meeting basic needs - Exemplary parents - Positive peer influence - Small families	- Absence of parental supervision - Disobedient adolescents - Negative peer influence - Abuse at home/Domestic violence - Negative parental influence
Community and Society factors	- Community and radio SRH programs - Role models - Child protection services	- Unregulated information in the media (radio, TV, social media) - Desire for material/monetary gains - Poverty- inability to meet needs - Negative community influence and norms - slums, acceptance of child marriage - Weak legal system - Widespread alcohol and drug abuse

### Protective factors

As shown in matrix 1, the study revealed protective factors at individual, relationship and broader community levels. At individual level, being in school, having personal goals/aspirations, use of contraceptives, access to SRH information and religion/fear of God were mentioned as key protective factors against adolescent pregnancy. Study participants viewed schools as keeping adolescents busy, offering guidance and counseling and promoting self-esteem and respect that keep adolescent away from engaging in premarital sexual activity thus avoiding becoming pregnant.

When a child is at school, she gets counseling from home and school. She comes back from school late and leaves home early to go to school. All the time she is busy that can help her to protect herself (FGD older out-of-school adolescents, Wakiso)

### Protective factors

As shown in matrix 1, the study revealed protective factors at individual, relationship and broader community levels. At individual level, being in school, having personal goals/aspirations, use of contraceptives, access to SRH information and religion/fear of God were mentioned as key protective factors against adolescent pregnancy. Study participants viewed schools as keeping adolescents busy, offering guidance and counseling and promoting self-esteem and respect that keep adolescent away from engaging in premarital sexual activity thus avoiding becoming pregnant.

*When a child is at school, she gets counseling from home and school. She comes back from school late and leaves home early to go to school. All the time she is busy that can help her to protect herself (FGD older out-of-school adolescents, Wakiso)*

Study findings revealed that adolescents who had good relationship with parents, received guidance and supervision and had their basic needs met were protected from pregnancy. Parent-adolescent communication including sexual and reproductive health was another protective factor against pregnancy.

*I know that when I get pregnant, I cannot return home. This is because whenever my father is counseling me, he tells me, “If you get pregnant you won't see me again.” I love him so much, so this message is ever on my mind. I'm not going to get pregnant.* (FGD older adolescents out-of-school, Wakiso)

*In families with few children, they (parents) give time to their adolescents but in families with many children, some are sent to the village to their grandparents who might not manage them. The rich usually have three or four children who they are able to cater for and also stay with them and they easily monitor them* (IDI pregnant adolescent Kamuli)

At community level, they cited availability of community support, education programs on SRH, counseling services and having good community role models like the Queen of Buganda, and the Speaker of Parliament who encourage adolescent girls to remain in school and avoid becoming pregnant.

Some children have their role models and she says I want to be like the other and she says if I do this and I dare get pregnant, then I can't manage becoming like her so she keeps herself in such a situation. Some want to be like the speaker of Palianament Hon Rebecca Kadaga, she also wants to be like her so she keeps saying I want to do exactly like what she did in other words it stays in her mind that I also have to walk in the right path and be able to reach where she is (FGD older out of school Kamuli)

### Factors increasing the risk of adolescent pregnancy

At individual level, inadequate information on SRH, early sexual debut, alcohol and drug abuse being out of school especially for older adolescents increased risks of becoming pregnant. Early sexual debut was another driver of adolescent pregnancy mentioned:

*Adolescents engage into sexual intercourse early. Here they are very sharp. They start at fourteen to fifteen years …. and remember that if a girl starts menstrual periods and indulges in sex, the end result is being pregnant* (IDI Adolescent, Kamuli)

At relationship level, absence of parental supervision, disobedient adolescents, negative influence from peers, child abuse and domestic violence as well as negative parental influence were key factors mentioned to increase adolescent pregnancy. Lack of parental supervision and guidance was mentioned as a factor leading to teenage pregnancy.

Negative influence of peers and parents were also mentioned as factors leading to adolescent pregnancy. It was noted that some girls are encouraged by their peers or parents to get into sexual relationships in anticipation of material or monetary gifts. Others become pregnant owing to negative influence from their peers.

*I could be with my friends and they share what they do with their boyfriends. One can tell you that they had fun and drunk (alcohol). This gives you ideas and you tell your boyfriend to do the same and the end result is pregnancy* (FGD Adolescents in school Wakiso)

At broader community level, unregulated information in the media (radio, TV, social media), desire for material/monetary gains among adolescents, poverty and inability to meet basic needs, negative community influence and norms such as acceptance of child marriage, widespread use of alcohol and drugs especially in slums and the weak legal and child protection system were key drivers of adolescent pregnancy in the study setting.

Most study participants attributed the high rates of adolescent pregnancy to unregulated media print and electronic media content that makes sexual activity attractive for young people and some try it out and become pregnant.

*You see even those films are vulgar (pornographic). A child may see a film that has sexual intercourse scenes in it and says that let me also try it out and she gets pregnant* (FGD older adolescents out of school Wakiso)

## Discussion

This study sought to determine risk factors for adolescent pregnancy among adolescent girls in one rural and one urban district in Uganda. Among 600 girls of whom 75% were in school, we found that independent risk factors for adolescent pregnancy were older age, out-of-school, having a friend who has ever had a teenage pregnancy, cigarette smoking experience, working for money and correct contraceptive knowledge. Protective factors included positive personal attitudes, higher education of the father and larger household size. The increased risk of pregnancy among older adolescents, and those who were out of school is likely because while they are in school, they are very engaged, receive guidance from school teachers and have a clear reason to avoid pregnancy. Those who are out-of-school are usually very poor and the increased needs coupled with little access to contraceptives predisposes them to pregnancy. Poverty has been noted to predispose to adolescent pregnancy^14^. Several studies included in systematic reviews have demonstrated that keeping adolescents in school is protective against adolescent pregnancy^2^,^15^.

Other independent risk factors for adolescent pregnancy identified by the study included having ever smoked and having a friend who had ever been pregnant. This reflects high risky behavior and peer influence which are both characteristic of the adolescent period. Risky behavior and peer pressure have been reported in a recent systematic review^16^. In a review of literature from Africa, having peers who had gone through pregnancy was a risk for repeat adolescent pregnancy^17^. Ochen et al also found that adolescents who reported experiences of intense peer pressure and taking alcohol had an increased likelihood of adolescent pregnancy^18^. Adolescents are likely to be influenced by their peers and take the practices of those around them therefore this finding is not surprising.

Having accurate knowledge on contraceptives increased the risk of pregnancy. This was a surprising finding. However, a recent study indicated the same finding^14^. It indicates that knowledge alone is not enough to prevent pregnancy among adolescents. It is also possible that they acquired the knowledge after getting pregnant. Maternal education has also been found to influence the outcomes of children in particular adolescent pregnancy.^2^ In fact it has been shown that where mothers had a pregnancy before the age of 19 years this increases the risk of adolescent pregnancy for their daughters^19^.

Living in a household size of 4-8 was found to be protective against adolescent pregnancy in comparison to smaller households. This is contrary to what has been reported in literature^14^. In our study, it is possible that the small families were those where adolescents were “married” with one child, making it a small household. Good personal attitudes or self-efficacy were protective against adolescent pregnancy. These adolescents believed it was not difficult for them to avoid a pregnancy. Good personal attitudes empower the adolescent to bargain and defend herself based on her belief system. This will protect her from the potential predaors and gives girls an upper hand to decide when to have sex, practicing protected sex and making decisions about using contraceptives. A recent study in Lagos among in-school adolescent girls reports that future orientation significantly influenced safe sex self-efficacy^20^.

Kamuli district in Eastern Uganda is predominantly a rural and poor population. Among the study population in Kamuli district, we found additional risk factors for adolescent pregnancy including: having been sexually abused, reported use of alcohol and engaging in work for money. Evidence suggests that adolescents living in rural settings are at higher risk of pregnancy compared to those living in urban settings^2^. This may be due to the effect of poverty in rural community. Sexual abuse and alcohol abuse are common in these settings that are poverty stricken.

In contrast, we found that amongst the adolescents in Wakiso district which is urban or peri-urban setting, those who had ever wanted to take their lives had a higher risk of teenage pregnancy. Depression and suicidal ideation have been shown to increase the risk of repeat adolescent pregnancy^17^. In Wakiso, coming from a rich family was protective against teenage pregnancy which was not in the rural setting. In urban areas, there is a clear difference in lifestyle and practices between the rich and the poor which may not be apparent in rural areas. Lower socio-economic status has also been found to be a risk factor for adolescent pregnancy^17^.

Furthermore, having discussed sex with one's parent/guardian, feeling safe while at home and good personal attitude were protective factors against pregnancy. Discussing matters regarding to sex between parents and adolescents has been found to be rare in Uganda and where this happens the discussion is likely to be unidirectional as opposed to open dialogue^21^. Discussing sex with the parents empowers the adolescent with information on pregnancy prevention^16^.

The strength of this study is that it employed both qualitative and quantitative methods of data collection. In addition, we captured data from a large number of adolescents, their parents, health workers, school teachers and other stakeholders.

## Conclusions

Overall, factors like older age, not going to school, low education of father, smaller household size, negative personal attitudes and accurate knowledge of contraceptives increased the risk of adolescent pregnancy among both urban and rural residing adolescents although the magnitude differs. There were some differences in risk factors between urban and rural adolescents, indicating the need for tailored interventions. Protective factors included having future goals and aspirations, good relationship with parents, positive peer influence and having role models.

We recommend that efforts to keep girls in school and to reduce poverty should continue since they are key in prevention of adolescent pregnancy. In addition, more support is needed to adolescents to improve their positive personal attitudes since it was protective in all adolescents. Contraceptive knowledge alone is not enough to prevent adolescent pregnancy. It has to go along with enforcing positive attitudes and self-efficacy among the adolescents. Parents need training on how to foster this. Furthermore, interventions to prevent adolescent pregnancy should be specific to the residence of adolescents. More research is needed to explore why accurate contraceptive knowledge was associated with adolescent pregnancy.
